# Viral Regulation of Prokaryotic Carbon Metabolism in a Hypereutrophic Freshwater Reservoir Ecosystem (Villerest, France)

**DOI:** 10.3389/fmicb.2016.00081

**Published:** 2016-02-09

**Authors:** Angia Sriram Pradeep Ram, Jonathan Colombet, Fanny Perriere, Antoine Thouvenot, Télesphore Sime-Ngando

**Affiliations:** ^1^UMR CNRS 6023, Laboratoire Microorganismes: Génome et Environnement, Clermont Université, Université Blaise PascalAubière, France; ^2^Athos Environnement, Université Blaise PascalAubière, France

**Keywords:** prokaryotes, viruses, seasonal dynamics, lytic infection, prokaryotic growth efficiency, Villerest reservoir

## Abstract

The current consensus concerning the viral regulation of prokaryotic carbon metabolism is less well-studied, compared to substrate availability. We explored the seasonal and vertical distribution of viruses and its relative influence on prokaryotic carbon metabolism in a hypereutrophic reservoir, Lake Villerest (France). Flow cytometry and transmission electron microscopy (TEM) analyses to determine viral abundance (VA; range = 6.1–63.5 × 10^7^ ml^-1^) and viral infection rates of prokaryotes (range = 5.3–32%) respectively suggested that both the parameters varied more significantly with depths than with seasons. Prokaryotic growth efficiency (PGE, considered as a proxy of prokaryotic carbon metabolism) calculated from prokaryotic production and respiration measurements (PGE = prokaryotic production/[prokaryotic production + prokaryotic respiration] × 100) varied from 14 to 80% across seasons and depths. Viruses through selective lyses had antagonistic impacts on PGE by regulating key prokaryotic metabolic processes (i.e., production and respiration). Higher viral lysis accompanied by higher respiration rates and lower PGE in the summer (mean = 22.9 ± 10.3%) than other seasons (mean = 59.1 ± 18.6%), led to significant loss of carbon through bacterial-viral loop and shifted the reservoir system to net heterotrophy. Our data therefore suggests that the putative adverse impact of viruses on the growth efficiency of the prokaryotic community can have strong implications on nutrient flux patterns and on the overall ecosystem metabolism in anthropogenic dominated aquatic systems such as Lake Villerest.

## Introduction

Heterotrophic prokaryotes are a prime biological component of the biosphere and account for a significant fraction of plankton biomass in aquatic systems. Factors that control their abundance and activity are crucial to understand their role in the biogeochemical cycles, the fate of organic carbon and nutrients, and the flow of energy to higher trophic levels (Azam and Malfatti, 2007). The two metabolic pathways in prokaryotes: synthesis of new biomass (i.e., prokaryotic secondary production, PP) and remineralisation of organic carbon to CO_2_ [prokaryotic respiration (PR)] are useful tools to understand the role of prokaryotes in ecosystem functioning. The relative magnitude of these two key metabolic processes (PP and PR) is controlled by prokaryotic growth efficiency (PGE), which is the fraction of assimilated organic C that supports biomass growth (del Giorgio and Cole, 1998). In aquatic systems PGE is considered to be the fundamental attribute of prokaryotic metabolism and strongly reactive to changes in the environment, yet the processes underlying this response is unclear (Smith and Prairie, 2004; Kritzberg et al., 2005; Maurice et al., 2010).

Prokaryotic growth efficiency was largely thought to be controlled by bottom–up factors (substrate supply), but with the recognition of viruses as dynamic components of planktonic communities in a wide variety of aquatic systems our conceptual understanding of the structural and functional organization of aquatic microbial food webs has changed (Suttle, 2007; Breitbart, 2012; Sime-Ngando, 2014). Growing evidence suggests that viruses are important agents which play vital role in the regulation of carbon and nutrient fluxes, food web dynamics and prokaryotic diversity (Fuhrman, 1999; Weinbauer, 2004; Hewson et al., 2010; Weitz and Wilhelm, 2012). In the recent years, increasing number of studies have focused on the viral regulation of PGE in both marine (Bonilla-Findji et al., 2008; Motegi et al., 2009; Xu et al., 2013) and freshwater systems (Maurice et al., 2010; Pollard and Ducklow, 2011; Pradeep Ram et al., 2015) but, however, with contrasting conclusions. Investigations on the viral infection on prokaryotic metabolism has not yet conclusive, as reports have suggested either negative effects of viruses on PGE (Motegi et al., 2009) or no effects (Xu et al., 2013). Viruses, as a regulator of bacterial growth, have been less well-studied, compared to substrate availability (Kirchman et al., 2004). The effect of viruses on PGE has been less well-understood than its effect on the prokaryotic community composition (Bouvier and del Giorgio, 2007; Sandaa et al., 2009). It is not known whether the influence of viruses on prokaryotic metabolism is the same across different trophic systems. Few quantitative data have been published on the above issue and many questions remain unanswered.

Freshwater reservoirs are complex systems where the disturbance frequencies are higher than in natural lakes with rapid often irregular and large, changes in flushing rates, water level, and the stability of the water column. They are thermally stratified systems which are characterized by natural physical feature that creates physicochemical gradients from surface waters to bottom sediments and forms anoxic zones especially during the warmer months (Pradeep Ram et al., 2009; Zhang et al., 2015). Reservoirs often receive substantial amount of anthropogenic (terrestrial) inputs from their drainage basin and watersheds.

Villerest Reservoir is a hyper-eutrophic and turbid lake reservoir (volume: 128 × 10^6^ m^3^, surface area: 30 km^2^, maximum depth: 40 m) located 5 km upstream from the city of Roanne, Central France. Since its construction in 1984 across the Loire River for the purpose of generating electricity by hydroelectric power plant, the reservoir has been characterized by an accelerated eutrophication caused by high nutrient loading, chiefly of phosphorus and nitrogen from the surrounding drainage basin (surface area: 130,000 km^2^) by human influences. Previous studies in this reservoir have reported high prokaryotic abundance and production rates; however, the fate of such production is uncertain. Since prokaryotes are the predominant host for viral proliferations and are known to impact prokaryotic community structure in aquatic systems, we hypothesize that viral infection should therefore exert a considerable and strong control on the PGE. The main aim of this study was to examine the impact of viral lytic infection cycle on their host carbon metabolism in a large artificial basin which is controlled by anthropogenic inputs. This study is of interest because there is limited data on the quantitative magnitude of the impact of viruses on prokaryotic metabolic processes such as prokaryotic production and respiration in stratified reservoir systems. In addition we also looked into the variation in the viral infection frequency and burst size (BS) for different prokaryotic morphopopulation in the water column.

## Materials and Methods

### Sampling

For detailed hydrological and morphometric characteristics of Villerest Reservoir see Jugnia et al. (2004). Sampling was carried out from April to November (2012) in the downstream part of the reservoir (45° 58′ 59 N, 4° 02′16 E). Water samples were collected bimonthly from May to October and monthly in April and November using a horizontal 10 L Van Dorn bottle. The sampling depths were located at epilimnion (0.5 m below the surface), metalimnion (10 m below the surface) and hypolimnion (1 m above the sediments), and are hereafter referred to as representative of these layers. According to Wetzel and Likens (1995), the Secchi depth measurement used to estimate euphotic depth did not exceed 6 m in the present study. After collection, water samples were immediately pre-filtered through a 150 μm pore size nylon filter to eliminate the predatory metazoan zooplankton and poured into clean recipients washed previously with lake water. Water samples were transported in dark and cold conditions within 2 h from the time of collection to the laboratory. All the samples were collected and analyzed in triplicates.

### Physico-Chemical Parameters

Water temperature and dissolved oxygen profiles were determined *in situ* using a WTW-OXI 320 multiparameter probe. Ammonia, nitrate, and orthophosphate were analyzed spectrophotometrically (APHA, 1998). Organic carbon concentrations namely total organic carbon (water sample passed through 150 μm filter) and dissolved organic carbon (water sample filtered through 0.7 μm pre-combusted glass fiber filter) were determined by high temperature catalytic oxidation method (680°C) using a TOC analyzer (Shimadzu TOC-V CPN, Japan; Lønborg and Søndergaard, 2009) and total dissolved nitrogen using the same analyzer with an attached measuring unit (Mahaffey et al., 2008). Concentrations of organic carbon and nitrogen were obtained from five point potassium hydrogen phthalate and potassium nitrate standard calibration curve respectively. All reported values were corrected for the instrument blank, and the CV was <5.0%. Water color was determined in filtered samples (0.45 μm) by reading absorbance at 440 nm using a Cecil CE2021 UV-Visible spectrophotometer (Cambridge, England) in a 10 cm cuvette (Pace and Cole, 2002). Samples were read against a blank prepared from nanopure water. Color is expressed as a wavelength specific absorption coefficient in units of inverse meters. Chlorophyll *a* concentrations (Chl) were determined spectrophotometrically from samples (500 ml) collected on Whatman GF/F filters. Pigments were extracted in 90% acetone overnight in the dark at 4°C, and concentrations were calculated from SCOR-Unesco (1966) equations. Chlorophyll values were converted to phytoplankton carbon using a value of 50 (Cho and Azam, 1988).

### Abundances of Viruses and Prokaryotes

Samples (1 ml each) for counting abundances of prokaryotes (PA) and viruses (VA) were fixed with glutaraldehyde (0.5% final concentration) and kept in dark at 4°C for 30 min. Abundances were determined using a FACS Calibur flow cytometer (Becton Dickinson, Franklin Lake, NJ, USA) equipped with an air-cooled laser providing 15 mW at 488 nm with the standard filter set-up as described by Marie et al. (1999), Brussaard (2004), and Duhamel and Jacquet (2006). Briefly, extracted samples were diluted with 0.2 μm prefiltered TE buffer (10 mM Tris-HCL and 1 mM EDTA, pH 8) and stained with SYBR Green I (10,000 fold dilution of commercial stock, Molecular Probes, Eugene, OR, USA). Mixture was incubated for 5 min, heated for 10 min at 80°C in the dark and cooled for 5 min prior to analysis. Prokaryotes and viruses differing in fluorescence intensity were detected by their signature in a side scatter versus green fluorescence (530 nm wave- length, fluorescence channel 1 of the instrument) plot. Flow cytometry list modes were analyzed using CellQuest Pro software (BD Biosciences, version 4.0). A blank was routinely examined to control for contamination of the equipment and reagents.

### Prokaryotic Growth Efficiency

The PGE was calculated from PR and PP measurements (Pradeep Ram et al., 2015). Briefly lake water samples were filtered through 1 μm polycarbonate (47 mm diameter) filters (Whatman, England) held in a Millipore filter holder using a peristaltic pump and silicone acid-washed tubing. Depending on the samples, the filters were replaced often to minimize the loss of prokaryotes due to clogging. This size fractionation procedure separated bacterioplankton from other planktonic components so that we could measure PR and PP with minimal interference from other planktonic organisms. The chosen pore size filters were effective and allowed over 85% of the free living prokaryotes to pass through, which was eventually confirmed by flow cytometry analysis of prokaryote abundances before and after filtration of water samples.

PR was estimated from the consumption of dissolved oxygen concentration in filtered water samples. For PR, six 150 ml capacity BOD bottles were carefully filled with samples. Three bottles were fixed immediately with Winkler’s reagents. Another three bottles were incubated in darkness at *in situ* temperature (±1°C) for 24 h before fixation. PR was determined from changes in dissolved oxygen by the Winkler method based on endpoint detection (Carignan et al., 1998). We used a factor of 0.375 to convert from oxygen to carbon units assuming a respiratory quotient of 1.

The prokaryote growth rate (μ) was determined by diluting the 1 μm-filtered water samples fourfold with 0.02 μm ultrafiltrate and incubated in the dark at *in situ* temperature (±1°C) over 24 h period. Prokaryotic cell increase was calculated by the equation for exponential growth: μ = (lnN_24__h_–lnN_0__h_)/T, where N_0__h_ and N_24__h_ represent prokaryote abundance at 0 and 24 h, respectively, and T is the incubation time. PP (cells l^-1^ h^-1^) was obtained by multiplying μ by the initial prokaryote abundance and then to carbon equivalents by using a conversion factor of 20 fg C per cell (Jugnia et al., 2000).

Prokaryotic growth efficiency was then calculated as PP/(PP + PR) and expressed in percentage (del Giorgio and Cole, 1998).

### Viral Lytic Infection and Viral Infected Prokaryotic Cell Morphotypes

Prokaryotic cells contained in formalin-fixed water samples (final conc. 2% v/v) were collected on triplicate electron microscope grids (400-mesh, carbon-coated Formvar film) by ultracentrifugation (Optima LE-80K, Beckman Coulter SW40 Ti Swing-Out-Rotor at 70,000 × *g* for 20 min at 4°C) according to Pradeep Ram and Sime-Ngando (2008). Each grid was stained at room temperature (ca. 20°C) for 30 s with uranyl acetate (2%, pH = 4), rinsed twice with 0.02 μm-filtered distilled water to remove excess stain, and dried on filter paper. The samples were examined using a JEOL 1200Ex TEM operated at 80 kV and a magnification of 20,000–60,000x to distinguish between prokaryotic cells with and without intracellular viruses. A prokaryote was considered infected when at least five viruses, identified by shape and size, were clearly visible inside the host cell. At least 400–600 prokaryotic cells were inspected per grid to determine frequency of visibly infected prokaryotic cells (FVICs). FVIC counts were converted to the frequency of infected cells (FICs) using the following formula: FIC = 9.524FVIC – 3.256 (Weinbauer et al., 2002) and thereafter to viral induced prokaryotic mortality (VIBM) using the following equation: VIBM = (FIC + 0.6FIC^2^)/1 -1.2FIC (Binder, 1999).

Viral infected prokaryotic cell morphotypes were subjectively recorded as elongated thin rod, short rod, fat rod, filamentous, and cocci (i.e., prokaryotic morphopopulations) based on observations during TEM examination. The above classification can, however, result in some overlaps among groups. To minimize this, cocci were defined as having a length: width ratio between 1 and 2, fat rods were defined as having a length:width ratio between 2 and 5 or having a width greater than 200 nm, and thin rods were defined as having a length : width ratio greater than 5 and a width less than 200 nm (Brum et al., 2005). BS was estimated from the number of viruses in those visibly infected cells that were totally filled with phages, i.e., the maximum BS (BSmax).

### Statistical Analyses

Differences in physicochemical and biological variables between seasons (Spring: April to June, Summer: June to September, and Autumn: September to November) were tested by one-way analysis of variance (ANOVA). Assuming that our triplicated samples for each sampling date and depth were independent replicates, we used two-way ANOVA to test the potential effects of seasons and depths on the variability in the targeted biological variables. Interactions terms were always included in the analyses. Potential relationships among variables (abiotic and biotic) were tested by linear pair-wise correlations (i.e., Pearson correlation analysis) and stepwise multiple regressions. Data were log transformed to satisfy the requirements of normality and homogeneity of variance necessary for parametric statistics. All statistical analyses were performed with Minitab software for Windows (Release 17, Minitab, State College, PA, USA).

## Results

### Physical Conditions

**FIGURE 1:**
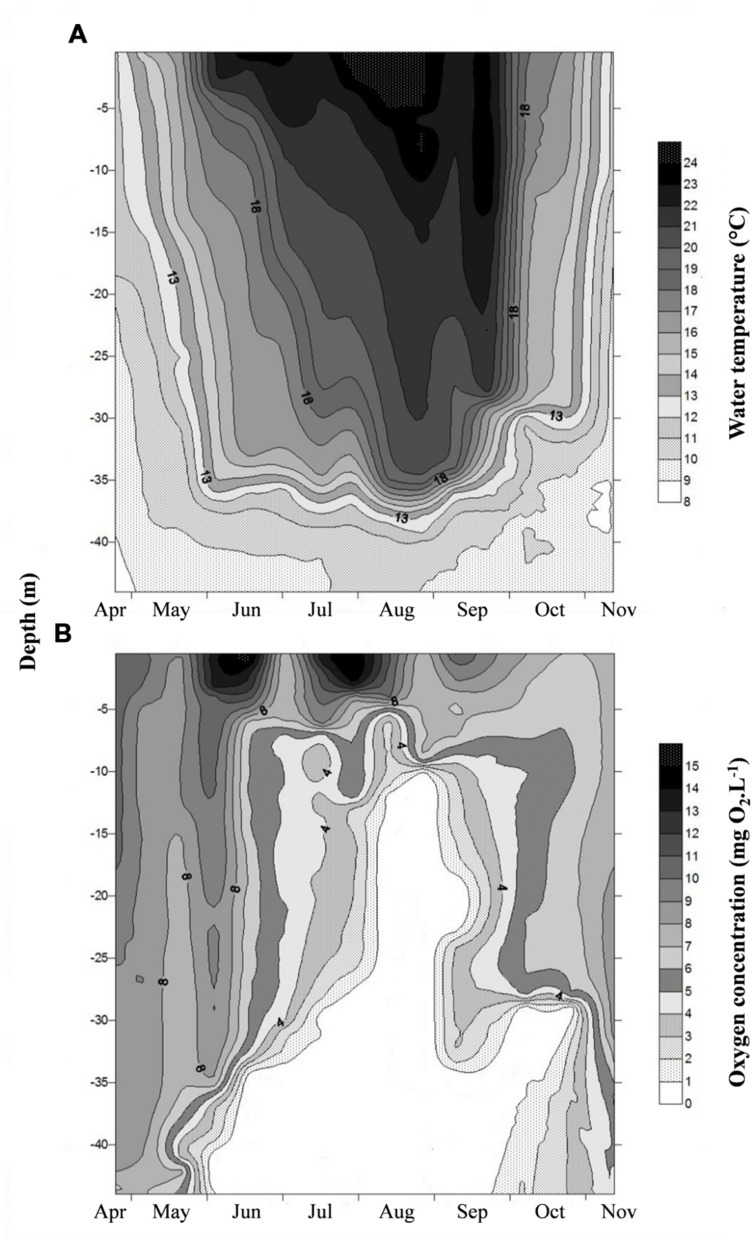
**Spatio-temporal variation of **(A)** water temperature and **(B)** dissolved oxygen concentration in the water column of Villerest reservoir from April to November 2012**.

Water temperature in the epilimnion (0.5 m depth) showed seasonal changes (*p* < 0.001); the values increased from 11.8°C (April) to 25.2°C (July) and then gradually decreased to 11.8°C toward end of study period in November (**Figure 1A**). A similar trend was also observed in the metalimnion but with lower values (**Table 1**). Water temperature in bottom waters (40 m) was low and less variable (8.9–10.9°C) throughout the investigation period. Thermal stratification started in early June and continued until late September. The water column above 10 m depth was generally well-oxygenated (>8 mg O_2_ l^-1^) during the entire study period, contrasting with the bottom waters where strong anoxic conditions prevailed from early June to late October (**Figure 1B**). Colored dissolved organic matter (CDOM) as determined from water color measurements was significantly higher (*p* < 0.001) in the bottom (range = 2.3–7.4 m^-1^) than in the surface waters (range = 1.2–2.9 m^-1^) due to intense degradation of organic particles largely made up of decaying phytoplankton cells. CDOM was strongly related to the water turbidity levels (2.4–10.9 ntu).

**Table 1:** Mean (range) environmental and microbial characteristics of the water column of Villerest Reservoir, April–November 2012.

Parameters	Epilimnion	Metalimnion	Hypolimnion
Temperature (°C)	20.2 (11.8–25.2)^a^	18.3 (11.7–23.5)^b^	10.4 (8.9–14.2)^ab^
Dissolved oxygen (mg l^-1^)^c^	10.5 (7.0–15.5)	5.9 (0.5–10.3)	2.1 (0.0–8.48)
NH_4_ – N (mg l^-1^)^c^	0.12 (0.05–0.30)	na	0.90 (0.05–2.2)
NO_3_ – N (mg l^-1^)	2.8 (0.20–5.7)	na	3.8 (1.1–7.8)
PO_4_ – P (mg l^-1^)^c^	0.07 (0.02–0.15)	na	0.32 (0.02–0.87)
Total organic carbon (mg l^-1^)	9.0 (5.7–12.7)	8.5 (6.0–10.7)	9.0 (6.8–11.4)
Dissolved organic carbon (mg l^-1^)	7.6 (5.6–9.4)	7.9 (5.5–10.2)	8.3 (6.3–10.4)
Chlorophyll *a* (μg l^-1^)^c^	22.2 (2.2–92.6)	2.5 (0.7–7.8)	1.5 (0.7–3.3)
CDOM (m^-1^)	2.2 (0.2–4.1)^a^	2.5 (0.4–5.1)^b^	4.5 (0.5–7.3)^ab^
Viral abundance (10^10^ l^-1^)^c^	29.5 (6.1–63.5)	17.6 (8.1–31.0)	13.7 (8.1–29.2)
Prokaryotic abundance (10^10^ cells l^-1^)	2.4 (1.1–4.1)^ab^	1.9 (1.1–3.0)^a^	1.9 (0.9–4.5)^b^
Virus-to-prokaryote ratio	12.2 (3.2–22.4)	9.4 (4.9–12.0)	7.6 (5.4–10.6)
Prokaryotic production (μg C l^-1^ h^-1^)	10.1 (2.6–26.9)	8.8 (ud–23.4)	10.4 (0.8–25.5)
Prokaryotic respiration (μg C l^-1^ h^-1^)	11.2 (2.1–26.3)^a^	11.3 (7.1–20.9)^b^	2.0 (ud–10.5)^ab^
Prokaryotic growth efficiency (PGE)	48.7 (13.8–80.2)^a^	44.8 (27.0–68.4)^b^	15.7 (ud–70.1)^ab^
Frequency of infected cells (%)^c^	18.8 (10.7–32.0)	14.2 (10.1–21.5)	9.5 (5.3–14.8)


*na, No data available; ud, under detectable limits.*

*Pairs of ^a^ or ^b^ indicate significant differences between two of the three layers (*p* < 0.05).*

*^c^Significant differences between the three layers (*p* < 0.05).*

### Nutrients and Chlorophyll Concentration

The concentration of potentially limiting inorganic nutrients such as nitrate, ammonia and dissolved reactive orthophosphate were well above the threshold concentration to induce any kind of limitation for the growth of planktonic organisms (**Table 1**). Nitrate recorded the highest concentration at both epilimnion (mean = 2.9 ± 1.8 mg l^-1^) and hypolimnion (3.8 ± 1.8 mg l^-1^) throughout the study period. Significantly (*p* < 0.001) higher concentration of ammonia and phosphate in the hypolimnion than epilimnion was associated with P leaching processes from lake bottom sediments (**Table 1**). There was little variation in dissolved organic carbon (DOC) measured at different depths suggesting homogenous concentration in the water column with the values mostly ranging between 5.6 and 10.4 mg l^-1^ (**Table 1**). Overall DOC represented 82–99% of the total organic carbon. The obvious presence of high organic carbon in the water column was linked to the high turbidity and CDOM levels in Villerest Reservoir. Chlorophyll concentration in the epilimnion ranged widely from 2.2 to 92.6 μg l^-1^ (mean = 22.5 ± 26.5 μg l^-1^) and these values were significantly higher (*p* < 0.001) than metalimnion (mean = 2.5 ± 1.9 μg l^-1^) and hypolimnion (mean = 1.5 ± 0.7 μg l^-1^). Overall chlorophyll was correlated (*p* < 0.01) to water temperature at all depths and to DOC concentration at epilimnion only.

### Standing Stocks of Viruses and Prokaryotes

**FIGURE 2:**
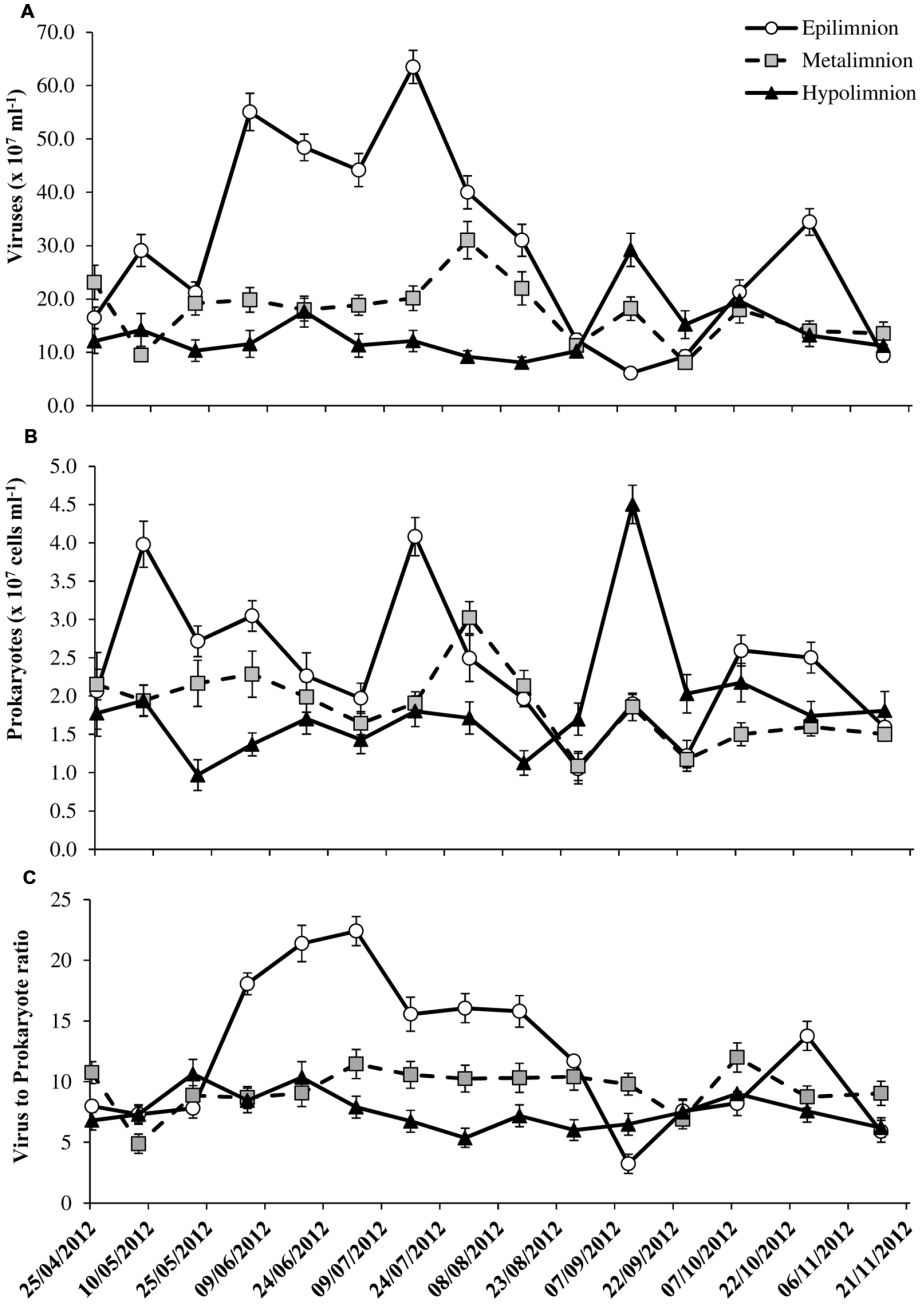
**Spatio-temporal variability in **(A)** viral abundance, **(B)** prokaryote abundance, and **(C)** virus to prokaryote ratio in the water column of Villerest reservoir, April–November 2012.** Error bars are SE.

VA were significantly higher (*p* < 0.001) and more variable in the epilimnion (mean = 29.5 ± 17.9 × 10^7^ ml^-1^) than at metalimnion (mean = 17.6 ± 5.8 × 10^7^ ml^-1^) and bottom layers (mean = 13.7 ± 5.3 × 10^7^ ml^-1^; **Table 1**). Two-way ANOVA indicated that VA exhibited strong variability with depth (*p* < 0.0001) than with seasons and both of which did not interact significantly (**Table 2**). Like VA, PA also showed similar trend with significant depth related variability (*p* < 0.003) than seasons (*p* > 0.05), but unlike VA both the factors interacted significantly (**Table 2**). In the water column PA and VA peaked on 16 July at epilimnion (VA = 63.5 × 10^7^ ml^-1^ and PA = 4.1 × 10^7^ cells ml^-1^) which was 10- and 4-fold higher than the lowest mean obtained for viruses (6.1 × 10^7^ ml^-1^) and prokaryotes (1.1 × 10^7^ cells ml^-1^; **Figures 2A,B**). Similar coincidence was also observed at metalimnion and hypolimnion on 30 July and 10 September respectively. Depth related variability in both PA and VA was due to the spatial difference that occurred during the period of thermal stratification (**Table 2**; **Figure 1**). Overall virus to prokaryote ratio (VPR) ranged from 3.2 to 22.4 (**Figure 2C**). High abundance and variability of viruses than prokaryotes led to significantly (*p* < 0.006) higher VPR at the epilimnion (mean = 12.2 ± 5.9) than hypolimnion (mean = 7.6 ± 1.5; **Table 1**). VA was significantly correlated (*p* < 0.02) to chlorophyll and PA in the epilimnion, and to PA only (*p* < 0.001) at meta and hypolimnion.

**Table 2:** Two-way ANOVA values for the effects of seasons and depths on viruses, prokaryotes, and frequency of infected cells in Villerest Reservoir.

Source	Viruses		Prokaryotes		Frequency of infected cells	
			
	*F*	*P*	*F*	*P*	*F*	*P*
Seasons (A)	1.55	0.216	1.16	<0.0001	2.57	0.081
Depths (B)	17.87	<0.0001	6.24	<0.003	28.13	<0.0001
Interactions (A × B)	0.92	0.453	3.37	<0.006	2.49	<0.04


*Degrees of freedom are 14, 2, 28, and 90 for A, B, AB and error, respectively. *F*, F ratio and *P*, probability.*

Forward stepwise multiple regression analyses suggested that for VA, both chlorophyll and PA were the main predictors for the oxic (VA = -14.78 + 1.30Chl + 10.62PA, *r*^2^ = 0.67, *p* < 0.001, *n* = 15) and, PA and DIN for anoxic bottom waters (VA = -0.17DIN + 5.86PA + 3.64, *r*^2^ = 82, *p* < 0.001, *n* = 15).

### Prokaryotic Production, Respiration, and Growth Efficiency

**FIGURE 3:**
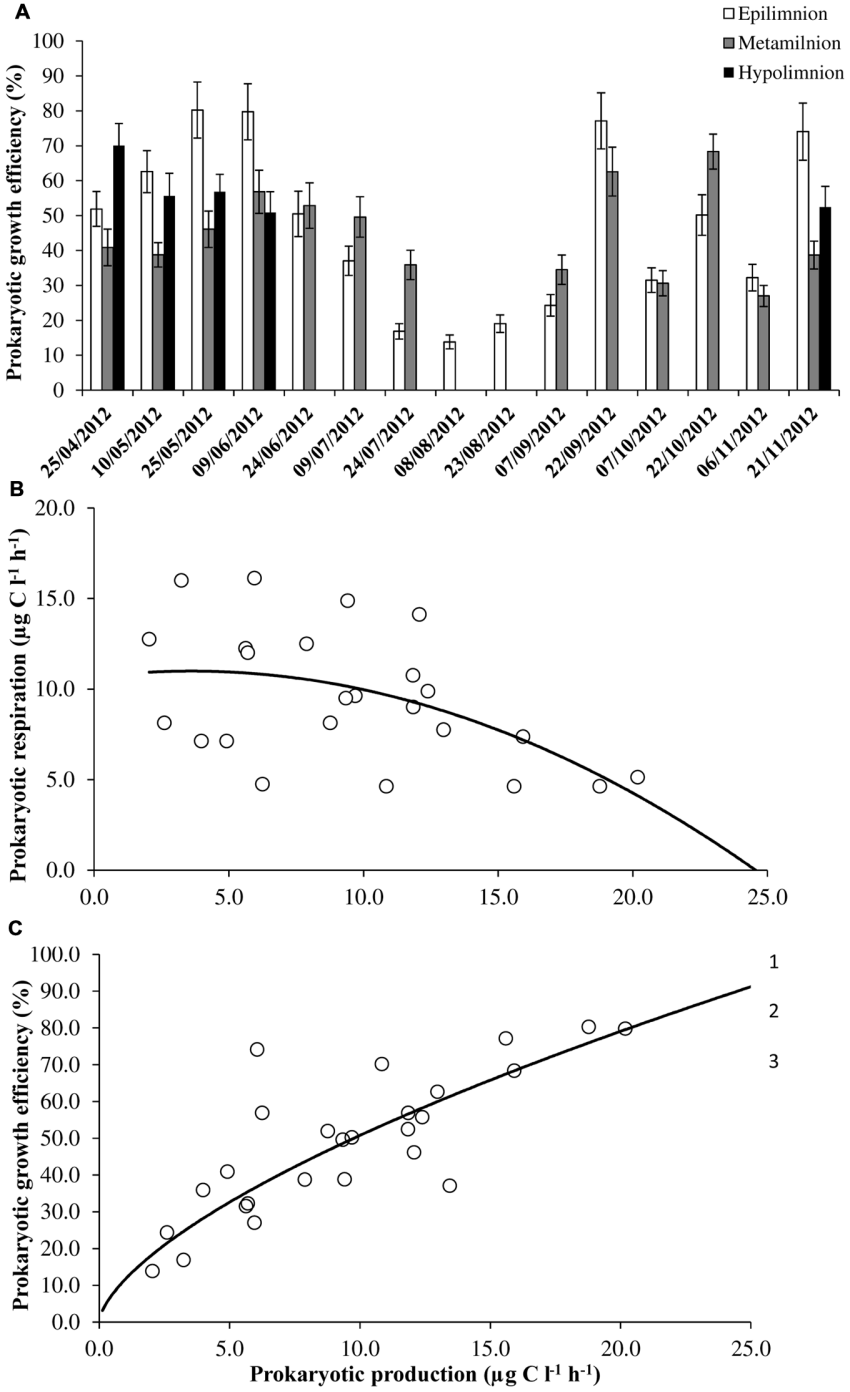
**Estimates of **(A)** prokaryotic growth efficiency, **(B)** the relation between prokaryotic production and respiration (PR = -0.03PP^2^ + 0.18PP + 10.67, *r* = -0.51, *p* < 0.01), and **(C)** prokaryotic growth efficiency and production (PGE = 11.63 × 0.64, *r* = 0.83, *p* < 0.001) in Villerest reservoir.** For seasonal variability and scatter plot diagram, data on PR and production (used to calculate prokaryotic growth efficiency) was not included during the period of anoxia that was observed in certain months at metalimnion and hypolimnion. Error bars are SE.

PP varied between 2.0 and 26.9 μg C l^-1^ h^-1^ and the values did not differ significantly between epi- (mean = 10.1 ± 7.7 μg C l^-1^ h^-1^) and hypolimnion (mean = 10.3 ± 5.8 μg C l^-1^ h^-1^; **Table 1**). Seasonal variability in PP was observed at epilimnion with significantly (*p* < 0.001) higher values in spring and autumn compared to summer months. At surface waters, high PP value was found to coincide with high DOC and ammonium concentrations. Overall PR ranged from 4.6 to 26.4 μg C l^-1^ h^-1^. Significantly, (*p* < 0.001) high PR values were observed in the summer months compared to spring and autumn at epilimnion. Strong anoxic conditions in majority of sampled months (July to October) at the hypolimnion and couple of months at metalimnion (August and September; **Figure 1**) prevented us from estimating the PR. Both PP and PR were assessed to determine variations in PGE and the sum of both the metabolic parameters (PR+PP) were used to determine prokaryotic carbon demand (PCD). The calculated PGE showed large variability especially at epilimnion which encompassed the entire range of values (14–80%) for the studied depths (**Figure 3A**). Over the range of reported data, negative correlation (*p* < 0.01) between PP and PR was observed only when PR was less than 20 μg C l^-1^ h^-1^ (**Figure 3B**) This uncoupling between PP and PR could perhaps be explained for the observed range in PGE values, as a consequence PGE was largely explained by the variation in PP (y = 11.63 × 0.64, *R*^2^ = 0.69, *p* < 0.001) at Villerest Reservoir (**Figure 3C**). Summer months were characterized by low PP and high PR values which led to comparatively lower PGE (mean = 22.9 ± 10.3%) than other months.

### Viral Lysis and Infection of Prokaryotic Cell Morphotypes

**FIGURE 4:**
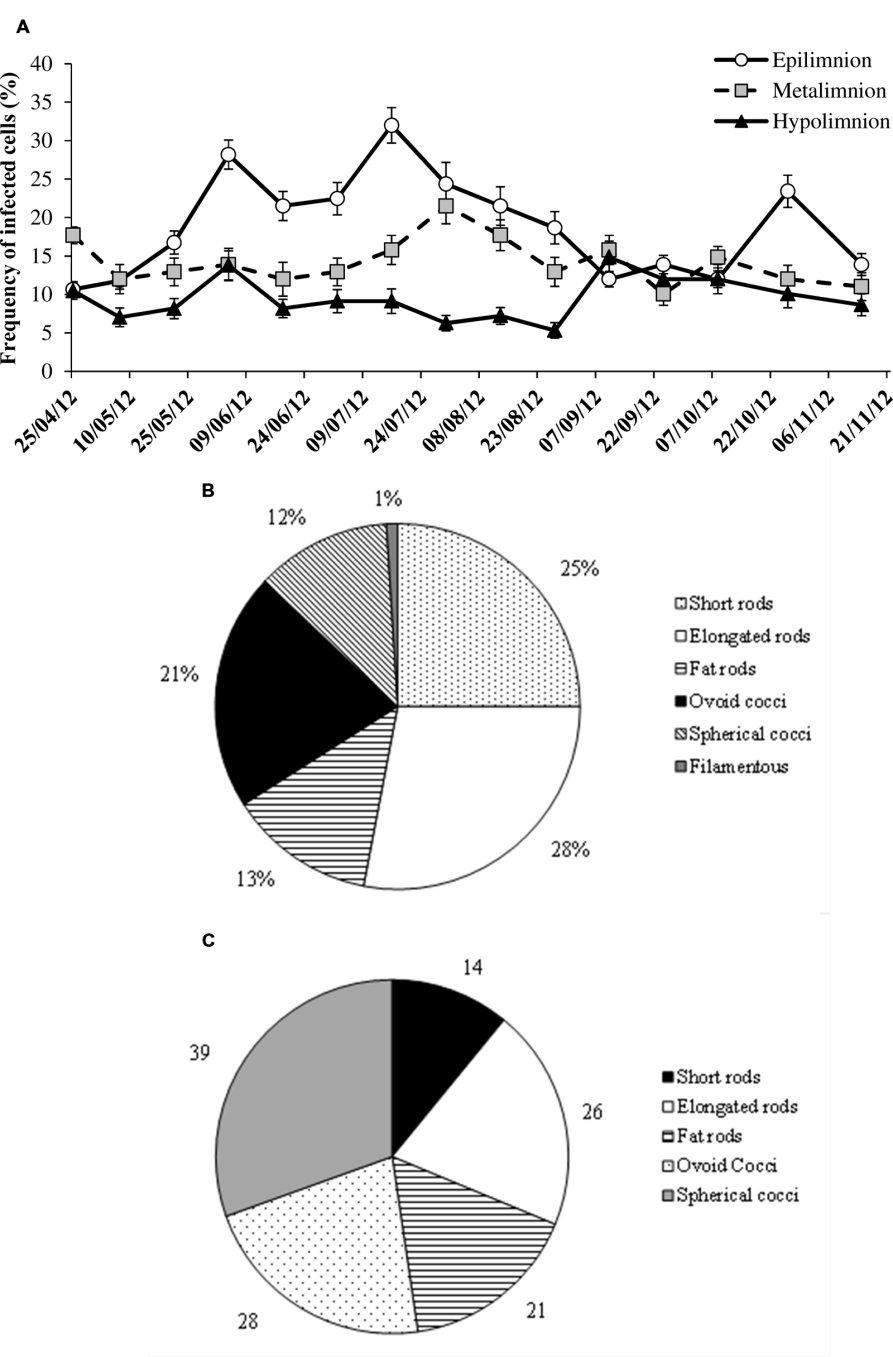
**Spatio-temporal variability in the **(A)** frequency of infected cells (FICs), **(B)** FICs (percentage of total infected cells), and **(C)** maximum burst size estimates (viruses per prokaryote) for different prokaryote morphotypes.** Error bars are SE.

Lytic viral infection, as determined from FVICs by TEM was detected at all the sampled occasions. Over the seasons and depths, lytic infection varied by a factor of 10 (**Figure 4A**). Overall the FICs varied from 5.3 to 32%, with a mean value of 14% that corresponded to 18% of the prokaryotic mortality caused by viruses (i.e., VIBM). Two-way ANOVA indicated that FIC exhibited strong variability (*p* < 0.0001) with depths than seasons and significant interaction (*p* < 0.04) between depths and seasons were observed (**Table 2**). The maxima in FIC at epilimnion coincided with peaks in PA and VA (**Figure 4**) and corresponded to a VIBM level of 62%. FIC at the epilimnion (mean = 18.9 ± 6.7%) was about twofold higher compared to hypolimnion (mean = 9.5 ± 2.7%). FIC was significantly correlated to abiotic variables such as water temperature and chlorophyll, and to standing stocks such as PA and VA at sampled depths. FIC was positively correlated (*p* < 0.05) to PR (**Table 3**). Regression analysis suggested that for FIC, a combination of chlorophyll, VA and temperature (FIC = 0.63Temp -0.37Chl + 0.31VA + 1.96, *r*^2^ = 0.81, *p* < 0.001, *n* = 15) and, VA and PA (FIC = -2.32PA + 0.71VA + 5.06, *r*^2^ = 68, *p* < 0.001, *n* = 15) were the main correlates at oxic and anoxic bottom waters respectively.

**Table 3:** Pearson product moment correlation (*r*) between different variables in Villerest Reservoir.

	Temp	DOC	Chl	VA	PA	FIC	PP	PR
DOC	NS							
Chl	0.62^∗∗∗^	NS						
VA	0.47^∗∗^	NS	0.72^∗∗∗^					
PA	NS	NS	0.37^∗^	0.72^∗∗∗^				
FIC	0.66^∗∗∗^	NS	0.74^∗∗∗^	0.87^∗∗∗^	0.50^∗∗^			
PP	NS	NS	NS	NS	0.32	-0.44^∗∗^		
PR	0.49^∗∗^	0.39^∗^	NS	0.43^∗^	NS	0.40^∗^	NS	
PGE	-0.38^∗^	NS	NS	NS	NS	-0.75^∗∗∗^	0.83^∗∗∗^	-0.51^∗∗^


*Temp, temperature; DOC, dissolved organic carbon; Chl, chlorophyll; VA, viral abundance; PA, prokaryotic abundance; FIC, frequency of infected cells; PP, prokaryotic production; PR, prokaryotic respiration; PGE, prokaryotic growth efficiency.*

*Levels of significance: ^∗^*p* < 0.05, ^∗∗^*p* < 0.01, ^∗∗∗^*p* < 0.001, NS, not significant.*

During the investigated period, the observed six different prokaryote cell morphotypes infected by viruses were short rods, elongated rods, fat rods, spherical cocci, ovococci, and filamentous forms. Among the viral infected prokaryotic populations, rods were the dominant fraction (63%) than cocci (36%) morphotypes. Among rod morphotype, viral infected prokaryotes belonged to elongated (28%) and short rods (25%). Filamentous forms contributed to less than 2% of infected cells. The percentage of infected cells belonging to different morphotypes is shown in **Figure 4B.** The prokaryote cells totally filled with matured phages defined as BS varied among different prokaryotic morphotypes with cocci and short rods recording the maximum and minimum BS respectively (**Figure 4C**).

### Viral Control of Prokaryotic Growth Efficiency

**FIGURE 5:**
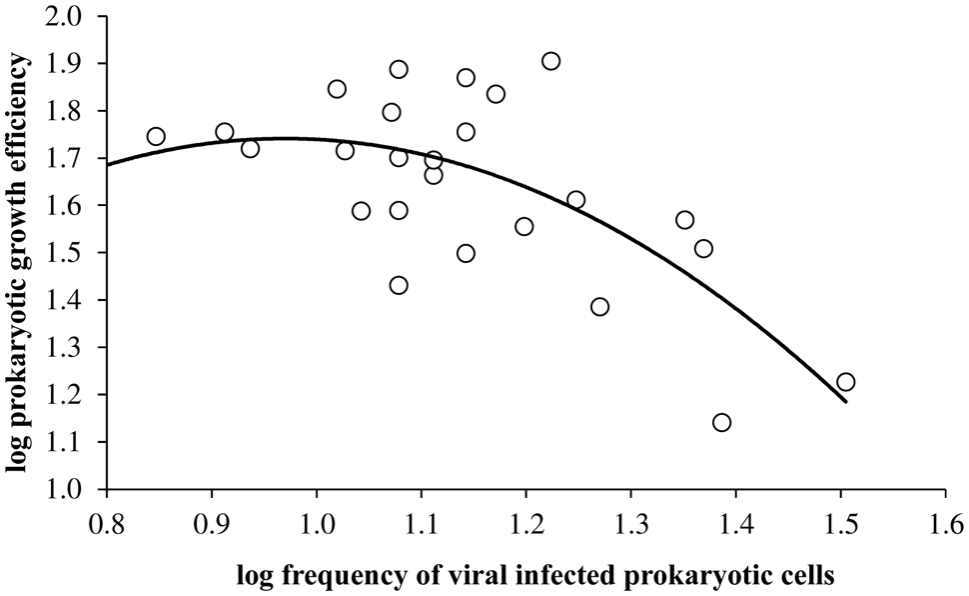
**Relationship between viral infected prokaryotic cells and prokaryotic growth efficiency (log PGE = -1.94 × log(FIC)^2^ + 3.77 × log(FIC) + 0.09, *r* = -0.69, *p* < 0.001) in Villerest reservoir**.

Overall, viral mediated prokaryotic mortality using the model of Binder (1999) ranged between 5.6 and 62% with higher mortality rates in the epilimnion than other sampled depths in the Villerest reservoir. In the epilimnion high mortality rates especially in the summer months (mean = 42 ± 12%) coincided with low PGE values (mean = 22.9 ± 10.3%). Consequently, viruses through its infection had adverse impact on PGE (log PGE = -1.94 × log(FIC)^2^ + 3.77 × log(FIC) + 0.09, *r* = -0.69, *p* < 0.001). The negative relationship between PGE and FIC was stronger and more significant when the FIC was greater than 10%. Differential relationship of viral infection with PGE could be best described by a concave quadratic regression model (**Figure 5**).

## Discussion

The present investigation is one of the few that reveals some notable features regarding the potential links between viral infection and prokaryotic community metabolism (referred to as PGE) in addition to environmental factors affecting the seasonal standing stocks of viruses and lytic phage infection in the water column of hyper eutrophic Villerest reservoir. Our findings indicate that viruses through their mortality processes had a putative adverse impact on the overall growth efficiency of prokaryotic community, which consequently can have strong implications on the carbon metabolism and fluxes in this artificial reservoir system.

### Methodological Aspects

In order to have a realistic estimation of PGE in Villerest reservoir, simultaneous measurements of PP and PR by size fractionation combined with short term incubation (24 h) was adopted (Kritzberg et al., 2005; Maurice et al., 2010; Pradeep Ram et al., 2013). This approach was found sufficient to detect linear decrease in dissolved oxygen concentration with concomitant increase in PA for the determination of PR and PP respectively. The advantages and limitations of using the above approaches have been taken into account and discussed in details elsewhere (Massana et al., 2001; Gattuso et al., 2002; Pradeep Ram et al., 2015). In spite of all precautionary measures such as the use of polycarbonate membrane filters instead of coarse glass fiber and low pressure filtration we cannot exclude the possibility that our respiration data might have errors due to artificial increase in labile DOM due to rupture of fragile cells.

TEM based derivation of FVIC combined with models of Binder (1999) and Weinbauer et al. (2002) to calculate VIBM has been increasingly used in recent studies conducted in freshwater lakes (Parvathi et al., 2014; Zhong et al., 2014; Pradeep Ram et al., 2015). We note also that the model used to derive VIBM from FVIC required several assumptions about the bacteriophage infection cycle, the parameters of which are still poorly constrained for populations in the environment (Binder, 1999). Although the above model has been utilized for wide range of systems with differing trophy, its applicability has not been validated in hyper eutrophic systems such as Villerest reservoir. Therefore, although the FVIC data were comparable to other studies, the estimates of viral mortality can have certain degrees of uncertainty.

### Standing Stocks and Lytic Phage Infection

The values of VAs (mean = 29.4 ± 17.9 × 10^7^ ml^-1^) that we report in Villerest reservoir is among the highest reported for temperate freshwater systems (Weinbauer, 2004; Brum et al., 2005; Maurice et al., 2010; Zhong et al., 2014). The VPR did not fluctuate considerably (not more than sevenfold) over the studied period suggesting relatively constant level of virus production and loss (Pradeep Ram et al., 2009). At the surface oxic waters significant correlation of VA with chlorophyll and prokaryotes suggest that the variation in viral standing stock was equally explained by these two variables. At deeper aphotic depths the large fraction (76%) of the variability in VA was explained by prokaryotes alone suggesting that the role of photosynthetic organisms as viral hosts should be minimal.

The percentage of FVIC that was determined through direct observation using TEM was comparable (0.9–3.7%) to the typical range of those (i.e., <5%) reported for limnetic systems (Weinbauer, 2004; Pradeep Ram et al., 2014; Sime-Ngando, 2014). Significant depth related differences in viral infection with twofold higher infection (FIC) rates at the oxic surface compared to anoxic bottom waters probably reflect the dramatic changes in the availability of hosts and the factors that control their abundance and activity in these contrasting environments (DeBruyn et al., 2004; Gobler et al., 2008). The increase in viral infection with increasing VA and PA would suggest that the lytic mode of infection is important and that specific contacts between them should yield higher infection rates. But, however, in spite of higher standing stocks viral lysis appeared to make a modest contribution to the mortality of prokaryotes. Several possibilities could be reasoned for the observed scenario. In lakes with high VA, the low detection of visibly infected prokaryotic cells by TEM in spite of high PP could be related to the decline in the phage maturation periods (period where intracellular virions are visible) in more active cells (You et al., 2002). The length of the lytic cycle can vary with the level of activity of the prokaryotic community, which suggests that infection of highly active cells with short phage maturation periods could yield lower apparent proportion of infected cells. Therefore, the observed decline in infected cells would not reflect an actual decline in infection rates, but rather a shift in the length of the lytic cycle that prevents the accumulation of visibly infected cells. It is possible that episodes of high infection rates occurred more frequently in the lake, but were short- lived and thus poorly resolved by our sampling intervals. Relatively, low infection amid high contact rates suggests that very few contacts result in productive infections. This could occur if the microbial community is more diverse than other environments, or if a large proportion of each population is resistant to co-occurring viruses. As for the second possibility, it is highly likely that there are significant sub-populations of prokaryotes resistant to co-occurring viruses (Brum et al., 2005). Resistance develops readily in prokaryotic populations exposed to a lytic bacteriophage, which can result in the stable coexistence of host and virus at high abundance (Levin et al., 1977). This phenomenon has been repeatedly demonstrated in theoretical and experimental studies (Levin et al., 1977; Bohannan and Lenski, 2000).

Phages target the most metabolically active and dominant fraction of prokaryotic community, which may be directly linked to their size characteristics (Ferla and Leonardi, 2005). In the present study, the abundances of dominant prokaryotes belonging to the rod morphotype (which comprises 53% of the total prokaryotic populations) were well above the threshold level of 2 × 10^5^ cells ml^-1^, necessary for the occurrence of detectable phage infection in the plankton (Weinbauer and Peduzzi, 1994). We consider that selective lyses of cells belonging to a particular morphotype, i.e., rod cells in our case study, may induce substantial changes in the functional roles of natural prokaryotic community.

### Factors Controlling PGE and its Regulation by Viruses

The relation between respiration and production expressed in the term PGE is a very important characterization of the ecosystem function(ing) and has also been used an index of prokaryotic physiological and energetic status at community level (del Giorgio and Cole, 1998; Maurice et al., 2010). In this study, the observed variability in PGE (14–80%) is considered to be a common feature of natural prokaryotic assemblage and agrees well with the expected range of values reported for eutrophic systems (del Giorgio and Cole, 1998; Smith and Prairie, 2004; Pollard and Ducklow, 2011). Prokaryotic populations tend to maximize their use of DOC; the energy generated in this process is then allocated between growth and maintenance, depending on the physiological state of prokaryotes and the inorganic nutrients available for growth (Pollard and Greenfield, 1997). In more than 50% of our data, PR was lower than PP and the plots between them were highly scattered indicating that both metabolic parameters were strongly uncoupled. Uncoupling between PP and respiration is considered to be more advantageous to natural bacterioplankton as it provides metabolic flexibility necessary to cope with ever changing nutrient conditions that are more often encountered in lake ecosystems (del Giorgio and Cole, 1998).

In Villerest reservoir the high abundances of prokaryotes were accompanied by high rates of PR and PP suggesting rapid turnover of prokaryotic community which could be dependent on the concentration of microbiologically labile organic substrates in DOC pool. Low phytoplankton carbon accompanied by high PCD (sum of PP and PR) suggest that that the autochthonous primary production may not be sufficient to support prokaryotic biomass activity. Henceforth, phytoplankton production can only contribute a part of the source of the DOC driving a substantial amount of heterotrophic production. The low percentage contribution of phytoplankton carbon to DOC and relatively high constant level of DOC concentrations (8.8 ± 1.8 mg C l^-1^) in Villerest reservoir suggest that continuous supply of dissolved organic matter from terrestrial or allochthonous sources was more important to explain for high PP and PR and meet their carbon demand. Although *in situ* primary production was not estimated in this study, PR seems to exceed the published rates on primary productivity from Villerest reservoir especially in summer months (Michard et al., 1995) which can perhaps signal the prevalence of net heterotrophy where the reservoir system can act as a net source of carbon to the atmosphere. In our study, respiration measurements were not related to chlorophyll concentration suggesting that the primary production might not be nutrient limited, considering the high concentration of inorganic nutrients. But, however, positive relation of PR with organic carbon reflects in part that prokaryotic metabolism is associated with increased inputs of organic carbon thereby further indicating that allochthonous inputs of organic matter may supplement ecosystem metabolism.

Viruses through lytic infection had a strong impact on metabolic processes such as PR and PP which eventually led to adverse or antagonistic effects on PGE especially in the surface waters. High viral lysis and VPR ratio especially in the summer months was accompanied by low PGE could be due to preferential lysis of active members of prokaryotic community which tend to exhibit high growth rates and activity. Our previous studies carried out in a set of lakes from French Massif Central have reported that the highly active high nucleic acid prokaryotic subgroup not only contribute to the overall growth efficiency of prokaryotic community (Pradeep Ram et al., 2015) but also are more susceptible to viral infection (Pradeep Ram et al., 2014). Low PGE amidst of high viral lysis strongly suggests that viruses would substantially alter the ratio between active and less active fraction of prokaryotes, therefore, they should be considered as an important top–down factor when seeking to explain factors controlling prokaryotic metabolism in aquatic systems. In accordance with our previous study, quadratic regression analysis from present study reiterates the fact that a minimum threshold viral infection level (i.e., FIC) of 10% is required for viruses to exert its impact on PGE. Our conclusion finds support from other previous studies from marine systems where viruses have shown to depress PGE at the community level (Bonilla-Findji et al., 2008; Motegi et al., 2009; Xu et al., 2013). Viral lytic infection transforms microbial biomass into dissolved and particulate organic matter. While high labile cellular components are rapidly assimilated by non-infected prokaryote cells, the less labile particulate forms such as cell debris (colloids and cell fragments) are incorporated in to prokaryote biomass by the action of its exo-enzymes which are associated with high respiratory losses, resulting in less flow of carbon to higher trophic level (Middelboe et al., 1996; Fuhrman, 1999). Viral lyses of prokaryotic hosts contribute to the pool of DOC in aquatic systems. However, in reservoir systems such as Villerest which receive high allochthonous inputs, the relative contribution of viruses to DOC pool is not known. Therefore, the relationship is more complex and more interesting to see its effect on PP and PR. Additionally high respiration of prokaryotic community in the presence of viruses in our study are supportive of the role of prokaryotes as oxidizers of organic matter, hence, as CO_2_ producers (Pollard and Ducklow, 2011). Although it is evident from our study that viral infection had an effect on prokaryotic metabolism, insufficient information exists as to how viruses regulate PGE through selective lyses of certain members of prokaryotic community. In order to explain the above, experimental verifications (time series laboratory microcosms) has been initiated to directly determine the impact of viruses on prokaryotic metabolism and their physiology in relation to prokaryotic community composition in such freshwater systems dominated by anthropogenic inputs.

## Author Contributions

ASPR designed the work, analyzed, and conceptualized the results. JC performed counts of prokaryotes and viruses using flow cytometer. FP performed nutrient analysis. AT was in charge of collection of water samples and performed profile of dissolved oxygen and temperature on each sampling date. TSN was involved in planning, and interpretation of the data.

## Conflict of Interest Statement

The authors declare that the research was conducted in the absence of any commercial or financial relationships that could be construed as a potential conflict of interest.
